# Editorial: Revisiting Cross-Cultural Comparison on International Business: Challenges and Opportunities

**DOI:** 10.3389/fpsyg.2022.941217

**Published:** 2022-06-13

**Authors:** Feng Jiang, Su Lu, Rui Zhang, Ning Zhang

**Affiliations:** ^1^Department of Human Resources and Organisational Behaviour, University of Greenwich, London, United Kingdom; ^2^School of Applied Social Science, De Montfort University, Leicester, United Kingdom; ^3^Department of Psychology, Dickinson College, Carlisle, PA, United States; ^4^School of Public Health and The Second Affiliated Hospital, Zhejiang University School of Medicine, Hangzhou, China

**Keywords:** cross-cultural, international business (IB), reliability, validity, theory

Cultural psychology has flourished over the past several decades. As a burgeoning research field, it deepens our understanding of people's culturally embedded beliefs, values, and behavioral patterns, with far-reaching implications for international communication and business (for a review, please refer to Leung et al., 2005). Despite its achievements, well-established cultural theories are facing increasing challenges in the border-crossing world of international business and organizational management. To illustrate, an example is the mixed findings regarding the importance of job autonomy. It has been widely assumed that job autonomy is promoted and thus should yield positive outcomes in Western societies (e.g., North America, Australia), as it aligns with the cultural values of individualism and low power distance (Wu et al., 2015; Tripathi et al., 2018). Empirical findings and practical observations, however, show a complex picture that sometimes the East and South Asian such as Chinese, Japanese, and Indians benefit similarly or even more from job autonomy compared with Westerners (Li, 2019; Charoensukmongkol, 2022). In her celebrating remarks on 50 years of the *Journal of Cross-Cultural Psychology*, Best (2019) exhorted scholars to continue to evaluate and leverage cultural differences to improve international communication and intergroup relations. This collection is intended to take a step in this direction by focusing on the predictive power of existing cultural models and the potential of new constructs in unpacking cultural differences.

The editorial call for a special issue on revisiting cross-cultural comparison on international business resulted in a number of impressive submissions from researchers in relevant fields. This collection contributes to cross-cultural literature with data from more than 60 countries. In addition, it involves varied and sophisticated methodological approaches including both quantitative and qualitative analyses. Two research groups focused on validating instruments of cultural values. Specifically, one study investigating intercultural adaptation revisited three established scales, the Multicultural Personality Questionnaire, Cultural Intelligence Scale, and the Intercultural Adjustment Potential Scale, and uncovered a new latent structure underlying the combined tests, consisting of five dimensions, namely assertiveness, attentiveness to others, emotional robustness, caution, and self-awareness (Matsumoto and Hwang). Another study re-evaluated Hofstede's Values Survey Module (VSM). Using a large dataset across 57 countries, Gerlach and Eriksson observed that the country scores of VSM based on this sample were weakly correlated with those scores obtained in previous studies. Also, the reliability of the VSM was poor in general. These studies echo the question we raised in this Research Topic: how can we enhance the prediction precision of cultural models? There is a clear need to update measures or develop new constructs of cultural dimensions. Meanwhile, the other research groups highlighted new observations on international business, such as cross-cultural leadership (Vaughan-Johnston et al.), advertising (Liu and Liu), and impulsive buying (Sun et al.). Findings from the studies featured in this collection shed light on the effects of culture on consumer behaviors, business communications, transnational marketing, etc. Collectively, the featured research represents key areas as well as identifies problems embedded in the current cultural models. However, much less is known about what kind of new constructs can incrementally unpack cultural differences. How do new constructs affect international business systematically? What is the nomological network of the new constructs? This collection is only one attempt to enrich cross-cultural theorizing. We hope it can spur future studies in deepening our understanding of the evolving dynamics of cultural constructs, and their antecedents and consequences across a variety of domains such as international business and cross-cultural communication.

## Author Contributions

FJ and SL drafted the paper. RZ and NZ edited it. All authors contributed to the article and approved the submitted version.

## Funding

The work was supported by the National Natural Science Foundation of China (No. 71971225).

## Conflict of Interest

The authors declare that the research was conducted in the absence of any commercial or financial relationships that could be construed as a potential conflict of interest.

## Publisher's Note

All claims expressed in this article are solely those of the authors and do not necessarily represent those of their affiliated organizations, or those of the publisher, the editors and the reviewers. Any product that may be evaluated in this article, or claim that may be made by its manufacturer, is not guaranteed or endorsed by the publisher.
